# “Sentinel or accomplice”: gut microbiota and microglia crosstalk in disorders of gut–brain interaction

**DOI:** 10.1093/procel/pwad020

**Published:** 2023-04-19

**Authors:** Haonan Zheng, Cunzheng Zhang, Jindong Zhang, Liping Duan

**Affiliations:** Department of Gastroenterology, Peking University Third Hospital, Beijing 100191, China; Beijing Key Laboratory for Helicobacter Pylori Infection and Upper Gastrointestinal Diseases, Beijing 100191, China; Department of Gastroenterology, Peking University Third Hospital, Beijing 100191, China; Beijing Key Laboratory for Helicobacter Pylori Infection and Upper Gastrointestinal Diseases, Beijing 100191, China; Department of Gastroenterology, Peking University Third Hospital, Beijing 100191, China; Beijing Key Laboratory for Helicobacter Pylori Infection and Upper Gastrointestinal Diseases, Beijing 100191, China; Department of Gastroenterology, Peking University Third Hospital, Beijing 100191, China; Beijing Key Laboratory for Helicobacter Pylori Infection and Upper Gastrointestinal Diseases, Beijing 100191, China

**Keywords:** gut microbiota, microglia, disorders of gut–brain interaction, irritable bowel syndrome

## Abstract

Abnormal brain–gut interaction is considered the core pathological mechanism behind the disorders of gut–brain interaction (DGBI), in which the intestinal microbiota plays an important role. Microglia are the “sentinels” of the central nervous system (CNS), which participate in tissue damage caused by traumatic brain injury, resist central infection and participate in neurogenesis, and are involved in the occurrence of various neurological diseases. With in-depth research on DGBI, we could find an interaction between the intestinal microbiota and microglia and that they are jointly involved in the occurrence of DGBI, especially in individuals with comorbidities of mental disorders, such as irritable bowel syndrome (IBS). This bidirectional regulation of microbiota and microglia provides a new direction for the treatment of DGBI. In this review, we focus on the role and underlying mechanism of the interaction between gut microbiota and microglia in DGBI, especially IBS, and the corresponding clinical application prospects and highlight its potential to treat DGBI in individuals with psychiatric comorbidities.

## Graphical Abstract

**Graphical Abstract:**
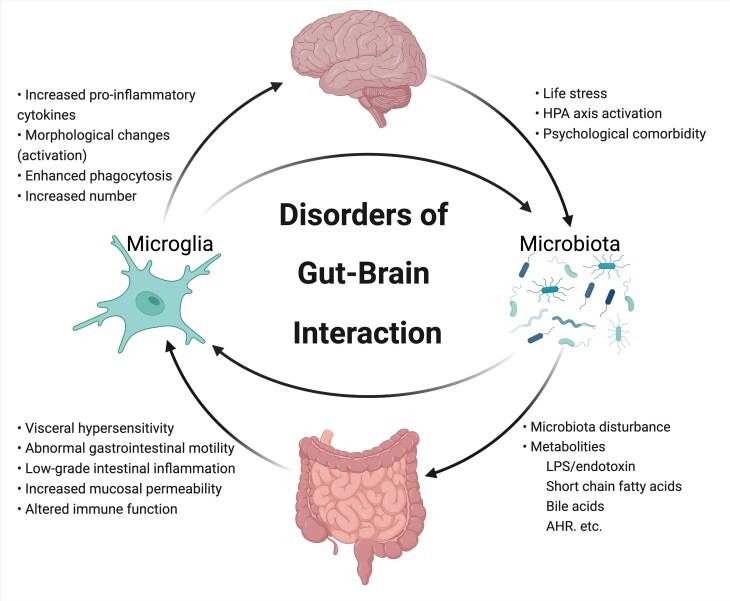

## Introduction

Disorders of gut–brain interactions (DGBIs), formerly known as functional gastrointestinal disorders (FGIDs), is a general term for a series of gastrointestinal (GI) symptoms without underlying structural abnormalities (Black et al., 2020a; Sperber et al., 2021). The three representative DGBIs are irritable bowel syndrome (IBS), functional dyspepsia (FD), and functional constipation (FC). A large-scale multinational study has shown that > 40% of people worldwide suffer from DGBIs (Sperber et al., 2021). Several clinical epidemiological studies have also found that patients with DGBIs have a high proportion of psychiatric comorbidities, such as anxiety and depression (Fond et al., 2014). These comorbidities lead to difficulties in the treatment of DGBIs and affect the quality-of-life and social functioning of patients (Sperber et al., 2021).

The pathophysiology of DGBIs is complex and diverse (Drossman and Hasler, 2016). Visceral hypersensitivity, abnormal GI motility, and psychological disorders are thought to be the main mechanisms that lead to DGBIs. Later, low-grade intestinal inflammation, increased intestinal mucosal permeability, altered immune function, and disturbances of the intestinal microbiota were also discovered to be involved in DGBIs. Based on this mechanism, the microbiota–gut–brain axis was proposed.

The gut microbiota of patients with DGBIs, particularly IBS, has been extensively studied (Simrén et al., 2013; Jalanka-Tuovinen et al., 2014; Lembo et al., 2016; Duan et al., 2019; Saffouri et al., 2019; Liu et al., 2022). Changes in the structure of the gut microbiota are associated with low-grade inflammation, such as mast cell activity in the colonic mucosa and elevated levels of colonic interleukin (IL)-12 in patients with IBS. In addition to low-grade intestinal inflammation, systemic inflammation was observed in these patients, manifested by elevated IL-12 levels and a decreased IL-10/IL-12 ratio in peripheral blood (Liu et al., 2022). Systemic inflammation can also lead to central nervous system (CNS) inflammation. Further studies using functional magnetic resonance imaging (fMRI) have found that patients with DGBIs have different degrees of activation of brain regions (Wilder-Smith, 2011), such as the medial prefrontal cortex, amygdala, and hippocampus. In addition, there was a significant correlation between IBS and the incidence of Alzheimer’s disease (AD) (Fu et al., 2020), Parkinson’s disease (PD) (Lu et al., 2022) and dementia (Chen et al., 2016). Mendelian randomization studies also found a significant genetic correlation between IBS and AD (Adewuyi et al., 2022). The gut microbiota is an important factor that affects the gut–brain axis. The symptoms of IBS can be improved through interventions targeting the gut microbiota, including probiotics (Zhang et al., 2022a), prebiotics (Ford et al., 2014; Huaman et al., 2018), low fermentable oligosaccharides, disaccharides, monosaccharides, and polyols diets (low-FODMAP diets) (Staudacher et al., 2014; Huaman et al., 2018), non-absorbable antibiotic rifaximin (Pimentel et al., 2011) and fecal microbiota transplantation (FMT) (Johnsen et al., 2018; El-Salhy et al., 2020).

Microglia are the resident immune cells of the brain that play key roles in a variety of neurodevelopmental processes required for proper brain maturation and function, such as neurogenesis, synapse shaping, and defense against infection. Changes in the structure and function of microglia are associated with various psychiatric disorders, such as depression (Torres-Platas et al., 2014; Tay et al., 2017) and Alzheimer’s disease (AD) (Moonen et al., 2023). In addition to the role of microglia in the CNS, microglia are involved in GI function and diseases. For example, visceral pain is associated with microglial activation in an IBS-like rat model (Yuan et al., 2020). Efferent nerves of the brain regulate GI motility, secretions, and permeability. Owing to the important physiological and pathological roles of microglia in CNS diseases, microglia are indispensable for the regulation of the gut–brain axis.

The gut microbiota can influence the CNS through neuronal, endocrine, and immune pathways (Margolis et al., 2021). Meanwhile, the CNS may also affect the gut microbiota structure. Gut microbiota and CNS have potential interactions and may mainly act on microglia. This relationship may be an important mechanism for DGBIs and psychiatric comorbidities, and has potential to be a future therapeutic target. Here, combined with our previous studies and recent research progress, we review and discuss whether the crosstalk between the gut microbiota and microglia participates in the development of DGBIs, especially IBS, from both gut-to-brain and brain-to-gut perspectives.

## Abnormal microbiota–gut–brain interaction in patients with IBS

IBS is the most typical DGBI. Approximately 40%–60% of patients with IBS have symptoms of anxiety and depression. Approximately 50% of these patients have a history of trauma (Fond et al., 2014). Greater insular activation and reduced inactivation of the pregenual anterior cingulate cortex have been observed in patients with IBS during noxious rectal distension. During anticipation of rectal distension, non-hypersensitive patients with IBS had more activation in the right hippocampus than the controls (Larsson et al., 2012). Notably, patients with IBS have increased levels of systemic cytokines, which is manifested by increased expression of IL-10 and IL-12 in peripheral blood (Zhang et al., 2019a). Patients with IBS with psychiatric comorbidities are often the most difficult to treat. Tricyclic antidepressants are most effective in alleviating abdominal pain in patients with IBS (Black et al., 2020b).

Previous cross-sectional and longitudinal studies have demonstrated an altered gut microbiota structure and function in patients with IBS (Mars et al., 2020; Mujagic et al., 2022). However, the results of various studies are highly heterogeneous. Most studies have mainly focused on the structure of the microbiota, and it is difficult to summarizing their microbial characteristics. However, the mechanism by which the intestinal microbiota causes IBS symptoms has not yet been elucidated. A recent study used metatranscriptomics and metabolomics to conduct a multi-omic assessment of the intestinal microbiota function in patients with IBS and found that after a series of parameter adjustments, IBS was associated with a differential abundance of bacterial taxa, such as *Bacteroides dorei*. The transcript abundance of some bacteria was increased in IBS, such as *Eggerthella lenta* and *Blautia hydrogenotrophica*; however, it decreased in *Bilophila wadsworthia*, *Roseburia inulinivorans*, and *Bifidobacterium animals* (Jacobs et al., 2023). Particularly, the gut microbiota structure is similar at the phylum level between patients with IBS and depression (Liu et al., 2016). This can be understood to a certain extent, as the pathogenesis of IBS is associated with comorbidities of mental disorders.

A study by Bercik provided new evidence regarding a species of gut microbiota that contributes to abdominal symptoms in IBS. The aforementioned low-FODMAP diet can relieve the symptoms of patients with DGBIs, which may be related to the lower histamine concentration in the urine of patients with IBS (McIntosh et al., 2017). Mast cells in the intestinal mucosa contain large amounts of histamine and are thought to be involved in the development of visceral hypersensitivity (Wouters et al., 2016; Balemans et al., 2019; Perna et al., 2021). In addition to mast cells, certain strains of gut microbiota are important sources of histamine. A recent study had detected high enrichment of *Klebsiella aerogenes*, a bacterium carrying a histidine decarboxylase gene variant, in fecal samples from patients with IBS. This revealed that *Klebsiella aerogenes* is an important source of intestinal lumen histamine, which partly explains why enterobacteria cause IBS abdominal pain (De Palma et al., 2022).

Intervention of the gut microbiota is effective in improving abdominal symptoms in patients with IBS and relieving psychiatric symptoms and central inflammation. Intervention with mixed probiotics (*Bifidobacterium longum*, *Lactobacillus acidophilus*, and *Enterococcus faecalis*) has been shown to significantly relieve IBS symptom severity scale (IBS-SSS) scores in patients with IBS and diarrhea (IBS-D), relieve abdominal pain symptoms, and reduce plasma monocyte chemoattractant protein-1 (MCP-1) and IL-1β levels (Zhang et al., 2019a). Furthermore, depression and coexisting GI symptoms can be significantly improved by probiotic intervention (Tian et al., 2022). In one such study, *Bifidobacterium longum* NCC3001 could increase the quality-of-life score and reduce the depression score in patients with IBS. Moreover, fMRI analysis showed that, compared with placebo, the *Bifidobacterium longum* NCC3001 intervention group reduced response of brain regions to negative emotional stimuli, including the amygdala and fronto-limbic regions. In other words, *Bifidobacterium longum* NCC3001 reduces the tendency of depression in patients with IBS (Pinto-Sanchez et al., 2017).

**Figure 1.**
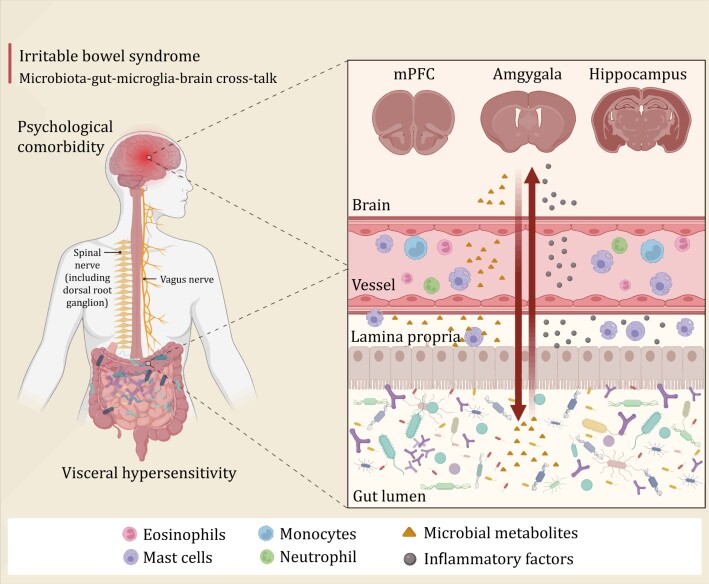
**Gut–brain interaction in irritable bowel syndrome**. Gut microbiota and its metabolites affect central nervous system function through neural, endocrine, and immune pathways. Disruption of the microbiota can lead to neuroinflammation that results in abnormal activation of multiple brain regions. Conversely, the activation of brain regions and neuroinflammation will release a variety of pro-inflammatory cytokines, which affect intestinal function and change the structure of intestinal microbiota through neural, immune, and endocrine pathways. Abnormalities in this gut–brain interaction collectively contribute to the comorbidity of psychiatric disorders in patients with IBS. mPFC, medial prefrontal cortex; IBS, irritable bowel syndrome.

In summary, abnormal brain–gut interactions are widely present in patients with DGBIs, which is the basis for the comorbidity of DGBIs and mental disorders. By regulating the intestinal microbiota, the “gut and brain co-treatment” can be partially realized, which is worthy of further discussion (Fig. 1).

## Microglia play an important role in gut–brain interactions

### Microglial properties and functions

Microglia are macrophages located in the brain parenchyma and spinal cord, and accounts for approximately 10% of all total CNS cells and 10%–15% of all glial cells. Unlike meninges and perivascular macrophages, microglia originate from the yolk sac and are derived from bone marrow-derived hematopoietic cells (Aguzzi et al., 2013). Microglial development can be divided into three phases based on gene expression: progenitor cell, embryonic, and mature. During the progenitor phase, microglia highly expresses genes involved in cell proliferation and DNA replication. The embryonic phase expresses genes related to nervous system development, intercellular signaling, and cell motility. The adult stage is characterized by involvement in cell development and immune activation (Matcovitch-Natan et al., 2016; Thion et al., 2018).

**Figure 2.**
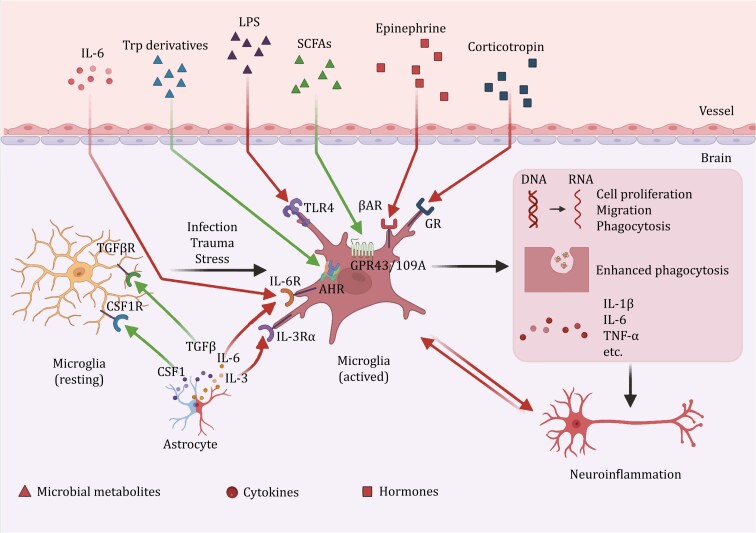
**Homeostasis and activation of microglia**. Resting microglia maintain homeostasis under the regulation of cytokines secreted by astrocytes and other cells. However, under conditions such as infection, trauma and stress, microglia are activated by various microbial metabolites, cytokines and hormones, leading to pro-inflammatory manifestations, causing neuroinflammation in the CNS and PNS. SCFAs and tryptophan derivatives play roles in inhibiting microglial activation. The red arrows represent pro-inflammatory pathways, and the green arrows represent mediators that inhibit inflammation or maintain normal growth and development of microglia. AHR, aryl hydrocarbon receptor; βAR, beta adrenergic receptor; CSF1, colony-stimulating factor 1; CSF1R, CSF1 receptor; GPR43/109A, G protein-coupled receptor 43/109A; GR, glucocorticoid receptor; IL, interleukin; IL-3Rα, IL-3 receptor alpha; IL-6R, IL-6 receptor; LPS, lipopolysaccharide; SCFAs, short-chain fatty acids; TGFβ: transforming growth factor beta; TGFβR, TGFβ receptor; TLR, toll-like receptor; TNF-α, tumor necrosis factor alpha; Trp, tryptophan.

Microglia are considered a part of the innate immune system in the brain and are known to exhibit a variety of morphologies. Resting microglia have small cell bodies and ramified processes that perform normal physiological functions. These microglia support the development and survival of neurons by releasing neurotrophic factors, removing dead cells from the CNS, preventing dysfunction in the CNS environment, regulating synaptic density and synaptic activity, and participating in the development of synaptic plasticity (Nayak et al., 2014). Amebic microglia appear in response to infection, trauma, mental stress, and central inflammation. Its cell body became larger with almost no processes, and the expression of cell proliferation, migration, and phagocytosis genes increased. Amebic microglia secrete high levels of inflammatory cytokines and mediate pathological pro-inflammatory, phagocytic, and immune-clearance functions (Wolf et al., 2017) (Fig. 2 and Table 1).

**Table 1.** Properties and functions of microglia.

Function of microglia	State	Action	Pathways and molecules	References
Neuronal support	Developing	Promote the development, and maintain the function of neurons	CX3CR1, IGF1, P2Y13	Araujo and Cotman (1992), Yamagata et al. (1995), Nakajima et al. (2001), Trang et al. (2011) and Ribeiro Xavier et al. (2015)
	Resting	Maintain the development and survival of neurons	BDNF, NGF, bFGF, EGF, HGF	Ziv et al. (2006), Ueno et al. (2013) and Stefani et al. (2018)
	Resting or activating	Monitor neural environment and response to damage	ATP, UTP	Davalos et al. (2005), Nimmerjahn et al. (2005) and Castellano et al. (2016)
	Stroke	Includes reduction of excitotoxic injury	NA	Szalay et al. (2016)
Neuronal damage	Developing	Promote neuronal apoptosis during development	NGF, CD11b, DAP12	Wakselman et al. (2008)
	Depression	Impair neurogenesis	IL-10, CX3CR1	Bassett et al. (2021) and Yang et al. (2021)
	AD	Cause damage or loss of neurons	CSF1R	Spangenberg et al. (2016) and Sosna et al. (2018)
	PD	Cause degeneration and depletion of dopaminergic neurons	CD200-CD200R, CX3CR1	Shan et al. (2011) and Zhang et al. (2011)
	Bacterial infection	Impair neurogenesis	TLR2, NO	Lehnardt et al. (2006) and Hoffmann et al. (2007a)
	Virus infection	Induce neuronal apoptosis	IFN-γ	Garber et al. (2019)
Phagocytosis	Developing	Regulate phagocytosis and shape CNS function	Endocannabinoid	Cunningham et al. (2013) and VanRyzin et al. (2019)
	Resting	Promote the clearance of apoptotic neurons	TREM2, DAP12, *Mertk*	Takahashi et al. (2005) and Damisah et al. (2020)
	AD	Mediate the phagocytosis of Aβ	CD47, TREM2,	Li et al. (2012), Zhao et al. (2018) and Dionisio-Santos et al. (2019)
	Bacterial infection	Increase phagocytosis activity against pathogens	TLR2, TLR4, TLR9	Ribes et al. (2010)
Synaptic pruning	Developing	Eliminate synapses to ensure proper brain connectivity	CX3CR1, complement, TREM2	Paolicelli et al. (2011), Filipello et al. (2018) and Gunner et al. (2019)
	Resting	Modify synaptic morphology	P2Y12, TWEAK	Tremblay et al (2010), Sipe et al. (2016) and Cheadle et al. (2020)
	Resting	Regulate synaptic function	ATP, THIK-1	Wake et al. (2009) and Badimon et al. (2020)
	Depression	Prune synapses in excess	complement	Li et al. (2023c) and Wang et al. (2023b)
	AD	Prune synapses in excess	CD47	Ding et al. (2021)
	Virus infection	Eliminate synapse Pathologically	IFN-γ	Garber et al. (2019)
Immune response	Depression	Secret IL-1β, IL-2, IL-6, TNF-α, and IFN-γ	NLRP3, PGE2	Jia et al. (2021)
	PD	Release TNF-α, NO, ROS	MMPs, LRRK2	Lee et al. (2010) and Moehle et al. (2012)
	Bacterial infection	Secrete pro-inflammatory cytokines, and chemokine	TLR1, TLR2, TLR4, TLR6	Prinz et al. (1999), Kielian (2004), Domínguez-Punaro et al. (2007) and Hoffmann et al. (2007b)
	Bacterial infection	Play the role of antigen presentation	MHC II, CD40, CD80, CD86	(Kielian T. 2004)
	Virus infection	Present the antigen (Moseman et al., 2020), and activate the T cells	MHC I, CSF1R	Wheeler et al. (2018) and Funk and Klein (2019)

ATP, adenosinetriphosphate; BDNF, brain-derived neurotrophic factor; bFGF, basic fibroblast growth factor; CX3CR1, C-X3-C motif chemokine receptor 1; DAP12, DNAX activation protein of 12 kDa; EGF, epidermal growth factor; HGF, hepatocyte growth factor; IFN, interferon; IGF1, insulin like growth factor 1; IL, interleukin; LRRK2, leucine-rich repeat kinase 2; MHC, major histocompatability complex; MMPs, matrix metalloproteinases; NGF, nerve gowth factor; NLRP3, pyrin-domain-containing 3; NO, nitric oxide; PGE2, prostaglandin E2; THIK-1, TWIK-related halothane-inhibited K channel; TLR, toll-like receptor; TREM2, triggering receptor expressed on myeloid cells-2; TWEAK, TNF-associated weak inducer of apoptosis; UTP, uridine triphosphate.

### Gut microbiota affects microglia

#### Gut microbiota influences microglia development, maturation, and function

Recently, the role of the gut microbiota and their metabolites in microglial function and central and peripheral nervous system dysfunction has received extensive attention. The most direct evidence of this is the abnormal development and function of microglia in germ-free (GF) animals. A previous study has shown that GF mice display global defects in microglia, with altered cell proportions (Erny et al., 2015; Thion et al., 2018) and an immature phenotype, leading to impaired innate immune responses (Erny et al., 2015). This suggests that the intestinal microbiota play a substantial role in maintaining the normal development of microglia. Further studies have shown that microglial colonization in GF mice is altered in a time- and sex-specific manner. Specifically, GF mice showed increased microglial density and hyper-differentiation at the embryonic stage, and the microglial density in male brains was significantly higher than that in females; however, in adult females, it increased relative to males (Thion et al., 2018). Gut microbiota also influences the development and aging of microglia. Microglia from GF mice exhibit reduced expression of genes associated with inflammatory and defense responses (Matcovitch-Natan et al., 2016). Microglia in aged specific pathogen-free (SPF) mice showed markedly altered morphology compared to young SPF mice. However, no significant difference was observed in microglia between aged and young GF mice (Mossad et al., 2022). Similarly, a key feature of microglial aging is increased oxidative stress, manifested by elevated levels of intracellular reactive oxygen species (ROS) (Streit, 2006). Microglial oxidative stress and ROS expression significantly increased in aged SPF mice; however, this increase was less pronounced in GF mice (Mossad et al., 2022).

Depletion of the gut microbiota and reduced diversity of the microbiota also affect microglial maturation and function. In addition to their antibacterial effects, antibiotics cause “side effects” that disturb the intestinal microbiota. Antibiotic (ABX) treatment promotes the downregulation of homeostasis-related genes *P2ry12*, *P2ry13*, *Selplg*, *Gpr165*, and *Cst3* in the spinal microglial cells of superoxide dismutase 1 (SOD1) mice (an amyotrophic lateral sclerosis animal model), whereas neurodegenerative disease characteristic-related genes include the upregulation of *Apoe*, *Lgals3 bp*, and *Cst7*. Antibiotics induce the transition of microglia from a homeostatic to a pathogenic state in SOD1 mice (Cox et al., 2022). Additionally, microglial overactivation due to antibiotic depletion of the gut microbiota exacerbates multiple diseases. For example, antibiotic treatment can worsen herpes simplex virus type 1 (HSV-1) infection, causing herpes simplex encephalitis. HSV-1 infection causes mitochondrial dysfunction, and the downregulation of PINK1-PRKN inhibits mitophagy and activates microglia to promote inflammation. Consequently, the nicotinamide oxidation product of neomycin-susceptible bacteria can limit microglial activation, promote mitophagy, and inhibit inflammation (Li et al., 2022a, 2023a). Furthermore, an FMT from non-ABX-treated mice to ABX-treated male mice reduced cecal enlargement and restored microglial structure and morphology in ABX mice (Dodiya et al., 2019). More importantly, the use of antibiotics will affect microglia and have a profound impact on future generations. Antibiotic-induced maternal microbiota dysbiosis (MMD) led to dysfunction of the intestinal microbiota and their metabolites in pregnant mice, increased expression of microglial senescence genes *Trp53* and *Il1β*, and caused abnormal social behavior in offspring mice. Maternal supplementation with *Lactobacillus murinus* HU-1 alleviated MMD-induced abnormalities in microglia and social behavior in offspring (Lebovitz et al., 2019). This result has been confirmed in clinical trials showing that prenatal antibiotic exposure during fetal delivery may increase the risk of various postpartum infection incidents (Nakitanda et al., 2023). Therefore, whether it is a GF or an antibiotic-treated animal, the morphology and function of microglia will be disturbed, which fully illustrates the regulation of the intestinal microbiota in microglia. However, how such a large number of intestinal microbiota affect and regulate microglia still requires elucidation.

Indeed, the composition of the microbiota structure in certain disease states may be an important factor in disease development. AD-associated gut microbiota (characterized by enrichment in *Bacteroides spp.*) can upregulate pro-inflammatory polyunsaturated fatty acid metabolism through activation of the C/EBPβ/AEP pathway, enhancing microglial activation and aggravating neuroinflammation, thereby promoting AD pathology and cognitive impairment (Chen et al., 2022a). Similarly, when sleep-deprived mouse microbiota were transplanted into normal mice, the normal mice showed excessive activation of microglia, apoptosis of neurons in the hippocampus, and cognitive decline (Wang et al., 2023a).

However, in this state of disease, what role does the microbiota play in regulating microglia? The commensal microbiota can promote the antigen presentation of microglia by activating the Toll-like receptor (TLR) 4 signaling pathway to help the host resist viral infection (Brown et al., 2019). Intervention with heat-killed *Mycobacterium vaccae* reduced the basal levels of genes involved in microglial priming (*Nlrp3* and *Nfkbia*), blocked microglial priming in response to immune stress, and attenuated stress-induced anxiety-like behavior (Frank et al., 2018). Prebiotic intervention can inhibit related pro-inflammatory and neurotoxic signaling pathways in α-synuclein-overexpressing mice and upregulate the neuroprotective phenotype of microglia (Abdel-Haq et al., 2022).

**Figure 3.**
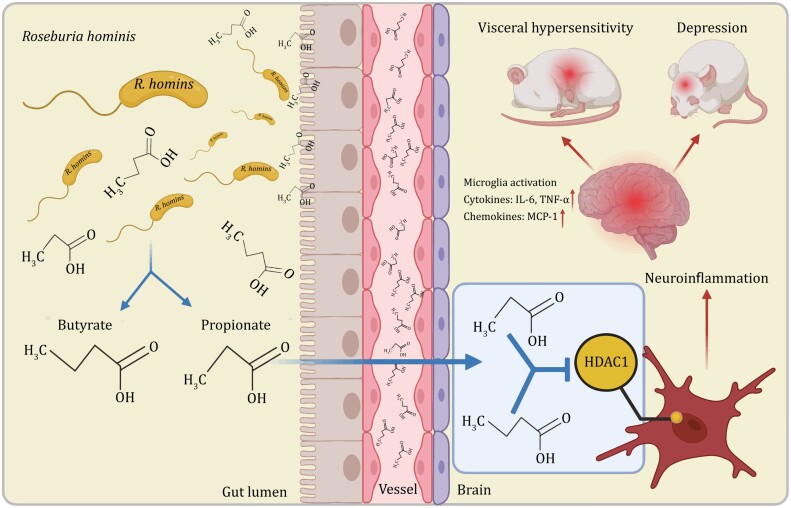
***Roseburia hominis* alleviates neuroinflammation in rat**. *Roseburia hominis* alleviates neuroinflammation by producing propionate and butyrate, which serve as HDAC inhibitors. This provides a potential psychoprobiotic to reduce neuroinflammation. HDAC1, histone deacetylase-1; IL-6, interleukin-6; TNF-α, tumor necrosis factor alpha.

It is a promising method for regulating microglia by regulating the intestinal microbiota to prevent and treat diseases. Therefore, understanding the effects of specific strains, such as the positive effects of known probiotics is urgently required. Various substances produced during the metabolism of the intestinal microbiota, such as short-chain fatty acids (SCFAs), are important factors that affect microglia (Silva et al., 2020). First, SCFA receptor G protein-coupled receptor (GPR43) (encoded by *Ffar2*)-deficient mice exhibit microglial defects consistent with those in GF mice (Erny et al., 2015). This suggests that SCFAs are important for microglial development and function. When we focused on one of specific SCFA, we found that microbiota-derived acetic acid can be taken up by microglia, and defects in gene expression, cell morphology, metabolic characteristics, and mitochondria of microglia in GF mice can be restored by supplementing acetic acid (Erny et al., 2021). *Clostridium butyricum* intervention improves motor function, inhibits microglial activation, and improves synaptic dysfunction in mice with Parkinson’s disease (PD) (Sun et al., 2021). Butyrate supplementation ameliorates chronic alcoholic CNS injury by inhibiting microglia-mediated neuroinflammation *via* the GPR109A/PPAR-γ/TLR4-NF-κB signaling pathway (Wei et al., 2023). Our study has shown that gavage with propionate and butyrate can improve abnormal microglial morphology in GF rats by inhibiting histone deacetylase-1 (HDAC1) expression and histone H3 acetylation K9 levels and reduce the expression of various inflammatory factors in the brain, such as those of IL-6, IFN-γ, and MCP-1 (Song et al., 2022) (Fig. 3). However, SCFAs released by microbiota also negatively affect the regulation of microglia. In a mouse model of AD, acetic acid inhibited the phagocytosis of amyloid-beta (Aβ) by microglia and increased Aβ plaques (Erny et al., 2021).

In addition to SCFAs, plant foods are metabolized by intestinal microorganisms to produce tryptophan derivatives, which activate the aryl hydrocarbon receptor (AHR) on microglial cells, directly promote transforming growth factor (TGF)-α, and inhibit the transcription of VEGF-β, thereby inhibiting the expression of the astrocyte inflammatory response and suppressing CNS inflammation (Rothhammer et al., 2018). Bile acid levels are also regulated by the gut microbiome. For microglia, activation of primary bile acid receptors such as sphingosine-1-phosphate receptor 2 can lead to activation of microglia and increased expression of chemokine ligand 2 release from neurons (McMillin et al., 2017; Agus et al., 2021). In contrast, secondary bile acids such as hyodeoxycholic acid and tauroursodeoxycholic acid may ameliorate CNS and microglial inflammation via the Takeda G protein-coupled receptor 5 (TGR5) and farnesoid X receptor (FXR) pathways (Bhargava et al., 2020; Zhu et al., 2022). Isoamylamine (IAA), a small-molecule metabolite produced by *Ruminococcus*, promotes microglial cell death and cognitive decline in aged mice by inducing S100A8 expression (Teng et al., 2022). Senescence of microglia manifests as ROS accumulation. Accumulation of ROS in the aging brain is associated with mitochondrial damage and mitochondrial dysfunction (Stefanatos and Sanz, 2018). Moreover, the intestinal microbiota can interact with intestinal epithelial mitochondria, affect mitochondrial function, and participate in the regulation of body homeostasis (Zhang et al., 2022b). Metabolomic analysis revealed that *N*6-carboxymethyllysine (CML), produced by bacterial metabolism, accumulated in the blood and brain of aged non-GF mice, and CML also increased with aging in human serum and brain. CML increases ROS levels and inhibits mitochondrial activity and adenosinetriphosphate (ATP) accumulation in microglia (Mossad et al., 2022). Branched-chain amino acids (BCAAs) are a group of essential amino acids, and the gut microbiota plays a role in regulating their levels (Agus et al., 2021). The accumulation of BCAAs caused by intestinal microbiota disturbance activates the AKT/STAT3/NF-κB pathway and exacerbates microglia-mediated neuroinflammation (Shen et al., 2023). The gut microbiome produces the metabolite trimethylamine, which is converted to trimethylamine *N*-oxide (TMAO) in the liver. High levels of TMAO increase the number of activated microglia and CNS inflammation (Li et al., 2022b). This process may be mediated by ROS production and the downregulation of methionine sulfoxide reductase A^76^ (Meng et al. 2019).

**Figure 4.**
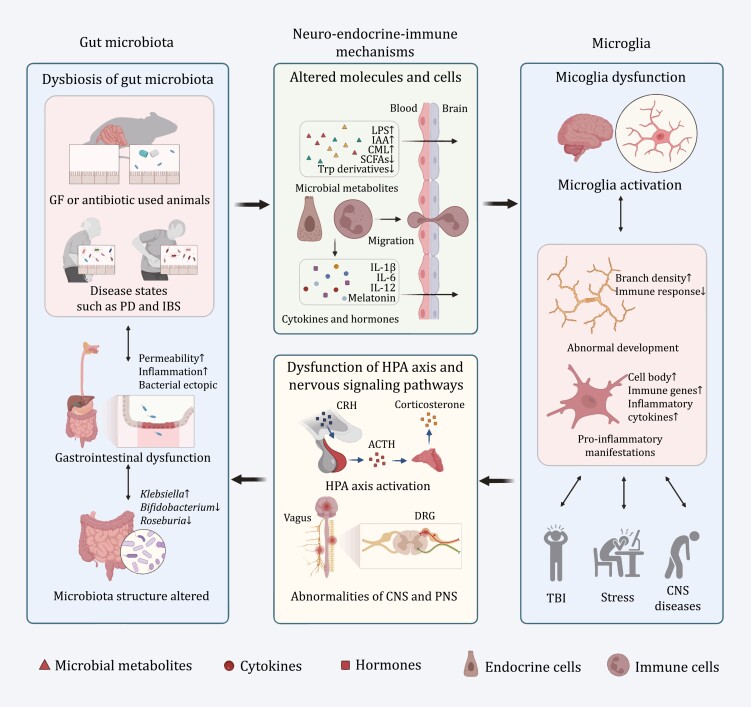
**Potential mechanisms of microbiota–microglia interaction**. In the absence or disturbance of the gut microbiota, the composition and abundance of microbial metabolites are altered. These metabolites, microbiota-derived inflammatory cytokines and hormones can enter the circulation and pass through the blood‒brain barrier, simultaneously with the migration of immune cells, leading to immaturity, abnormal activation and increased pro-inflammatory manifestations in microglia. Microglia are activated and participate in central inflammation and nervous signaling pathway dysfunction in brain injury, stress and a variety of psychiatric and neurodegenerative diseases, therefore causing HPA axis activation and abnormalities of the CNS and PNS. Then, GI dysfunction appears and further alters the dysbiosis of the gut microbiota. ACTH, adrenocorticotropic hormone; CML, *N*6-carboxymethyllysine; CNS, central nervous system; CRH, corticotropin-releasing hormone; DRG, dorsal root ganglion; GF, germ-free; IAA, isoamylamine; HPA, hypothalamic‒pituitary‒adrenal; IBS, irritable bowel syndrome; LPS, lipopolysaccharide; PD, Parkinson’s disease; PNS, peripheral nervous system; SCFAs, short-chain fatty acids; Trp, tryptophan.

We briefly summarized and exemplified some possible mechanisms by which the gut microbiota regulates microglia (Figs. 2 and 4).

#### Influence of gut microbiota on microglia in IBS

Although there is a lack of evidence for microglial activation in population cohort studies, various animal models of visceral hypersensitivity in IBS support this hypothesis. In animal models of IBS visceral hypersensitivity, the number of microglial cells in the dorsal horn of the spinal cord and brain increases, and is substantially activated (Zhang et al., 2016; Lucarini et al., 2020; Yuan et al., 2020; Atmani et al., 2022; Ji et al., 2022). The number of microglia in the dorsal horn of the L6-S1 spinal cord increased in a mouse model of visceral hypersensitivity, induced by intravesical perfusion of acetic acid (Atmani et al., 2022). The number of M1 pro-inflammatory microglia and M1 pro-inflammatory microglia-derived cytokines IL-6 and tumor necrosis alpha (TNF-α) increased in the anterior lateral bed nucleus of the stria terminalis of neonatal colorectal distension-induced chronic visceral hyperalgesia model rats (Ji et al., 2022).

Additionally, disturbances in gut microbiota have been reported in different IBS models. As previously mentioned, gut microbiota has three pathways affecting the CNS (Margolis et al., 2021).

For immune pathways, gut microbiota plays essential role in immune development and function (Belkaid and Harrison, 2017). For example, segmented filamentous bacteria (SFB) can adhere to the ileum and induce differentiation of T helper 17 cells (Ivanov et al., 2009). In addition, the gut microbiota can release a variety of metabolites and components such as lipopolysaccharide (LPS) and peptidoglycan into the circulating blood, thereby affecting the functions of the central and peripheral nervous systems (Agirman et al., 2021). The entry of gut-derived inflammatory factors (such as IL-1β, IL-6, and TNF-α) into the circulation may alter the integrity of the blood–brain barrier (BBB) and affect brain development (Erny et al., 2015; Thion et al., 2018; Parker et al., 2020). These metabolites and cytokines cause the activation of immune cells in the brain through the BBB, and can also activate immune cells in the intestinal tract. Some immune cells can even migrate to the CNS to promote or inhibit neuroinflammation and peripheral inflammation. Our previous studies have shown that transplantation of fecal microbiota from patients with IBS-D into GF rats can lead to visceral hypersensitivity and intestinal inflammation (as shown by an increased number of mast cells). Simultaneously, the spinal cord microglial cells of GF rats transplanted with feces from patients with IBS-D were significantly activated, and the branch area, length, number of branch points, number of segments, and number of terminal points were significantly lower than those in the control group (Zhang et al., 2021). *Roseburia hominis* gavage significantly reduces visceral hypersensitivity and improves microglial activation in the cingulate gyrus, dorsal hippocampus, and spinal cord of GF rats (Song et al., 2022).

For endocrine pathway, inflammation-induced activation of the hypothalamic–pituitary–adrenal (HPA) axis triggers the release of glucocorticoids, altering gut function (Cryan et al., 2019). The function of the HPA axis is regulated by the gut microbiota. Corticosterone levels and HPA axis activity were increased in GF mice, suggesting a central role for gut microbiota in HPA axis development and neuromodulation. Moreover, probiotics may affect neuroendocrine physiological homeostasis through immune regulation and reducing intestinal microbiota translocation (Cohen et al., 2015).

Melatonin is the main hormone in the pineal gland and is primarily synthesized by enterochromaffin cells in the GI tract. Melatonin regulates immunity, inflammation, and mood, relieves anxiety and depression, promotes sleep, and regulating GI motility (Ma et al., 2020). Patients with IBS-D have elevated melatonin levels and increased numbers of mast cells in the colonic mucosa. Melatonin may participate in the regulation of visceral hypersensitivity in IBS-D by inhibiting the activation of colonic mucosal mast cells. When GF rats received FMT from patients with IBS-D, the colonic mucosal melatonin levels increased. Further analysis revealed that *Roseburia* and *Lachnospira* species may promote colonic mucosal melatonin production through their metabolite butyrate (Wang et al., 2020). When GF rats were administered *Roseburia hominis*, melatonin levels in the ileum and colonic mucosa increased. Propionate and butyrate levels also increased. Furthermore, after gavage with propionic acid and butyric acid in GF rats, melatonin levels in the peripheral blood, ileum, and colonic mucosa also increased (Song et al., 2021).

For neuronal pathways, the most typical is the two-way regulation of gut microbiota and vagus nerve. *Campylobacter jejuni* can induce anxiety-related behaviors by enhancing vagal signaling to the nucleus tractus solitarius (NTS) (Goehler et al., 2005). Ameliorative effects of *Lactobacillus rhamnosus* JB1 on anxiety-related and depression-like behaviors can be blocked by vagotomy (Bravo et al., 2011). In turn the stimulation of afferent vagal and dorsal root ganglion (DRG) neurons that sense inflammation triggers CNS circuits involved in HPA axis activation, disease behavior [such as anorexia (Rao et al., 2017)], and visceral pain perception. Efferent nerves send signals to immune cells, inhibit the invasion of pathogenic bacteria, and induce the enrichment of protective microbiota by inhibiting pro-inflammatory macrophages, regulating M cell function (Lai et al., 2020), and inhibiting intestinal inflammation. A rat model of water avoidance stress (WAS) exhibited elevated visceral sensitivity, increased fecal particle counts, and activation of microglial cells in the dorsal horn of the spinal cord (Zhang et al., 2019b, 2021). Similar results have been observed in a mouse model of visceral hypersensitivity induced by chronic WAS (Yuan et al., 2020). Additional supplementation of *Roseburia hominis* can improve the abnormalities caused by WAS stress in rats, such as the decline of corticotropin-releasing hormone (CRH), reduction in visceral sensitivity, and decreasing of the fecal pellet number (Zhang et al., 2019b).

Correction or intervention of intestinal microbiota can inhibit microglial activation, relieve central inflammation, and improve IBS symptoms. The relative abundances of *Ruminococcus* and *Spirillum* increased when rifaximin was administered to animals with chronic and unpredictable mild stress (CUMS). In addition, rifaximin, a non-absorbable antibiotic, prevents and ameliorates CUMS-induced central functional abnormalities and elevates butyrate levels in the brain (Li et al., 2021). Some traditional Chinese medicine has been shown to have certain beneficial effects. Shugan granules are patented Chinese medicines. Shugan granule treatment in rats with chronic restraint stress can regulate intestinal microbiota, increase the abundance of *Bacteroides*, and improve the activation of microglia and intestinal barrier repair in model animals (Li et al., 2022c). Ginsenosides are the main active ingredients in ginseng and have a wide range of pharmacological effects. Ginsenoside Rh4 alleviates intestinal microbiota disturbances in depression-like mice, increases the content of SCFAs, inhibits excessive activation of microglia and astrocytes, and improves depressive behavior. Correlation analysis revealed that the Rh4-inhibited LPS/NLRP3/caspase-1/IL-1β signaling pathway plays a key role in improving depression (Shao et al., 2023). Berberine (BBR) is a natural alkaloid that exerts various physiological functions in the body by regulating the intestinal microbiota. BBR can significantly alleviate visceral hypersensitivity induced by chronic WAS and reduce the activation of colonic mast cells and spinal cord microglia by enriching SCFA-producing bacteria (Zhang et al., 2021). In addition, the psychbiotic *Roseburia hominis* inhibits the activation of microglial cells, reducing the level of inflammation in the brain, relieving neuroinflammation, and reducing depression-like behavior (Song et al., 2022) (Fig. 3).

### Microglia affect gut microbiota

#### Effects of the CNS on intestinal microbiota

Many studies have found that the CNS, especially microglia, can regulate the structure and function of intestinal microbiota. Traumatic brain injury (TBI) is an excellent candidate model for studying the brain–gut–microbiota axis as the earliest pathological change that occurs in the brain. TBI is accompanied by abnormal changes in microglia. In addition to central manifestations, patients with TBI have abnormal GI function with decreased GI smooth muscle contractility, delayed transit time, epithelial cell shedding, intestinal villus rupture, focal ulceration, and decreased expression of intestinal tight junction proteins such as occludin and zonula occludens-1. In TBI animal models, an imbalance in the intestinal microbiota of mice has also been observed. Specifically, the severity of TBI was related to the abundance of *Bacteroides*, *Porphyromonas*, Firmicutes, and Proteobacteria (Sundman et al., 2017). In TBI, these changes in the gut microbiota are directly caused by damage to the CNS and abnormal changes in microglia.

Central stress also induces changes in the gut microbiota and is involved in the activation of microglia through β-adrenergic signaling and the glucocorticoid receptor (GR) on microglia (Johnson et al., 2005; Blandino et al., 2006; Picard et al., 2021). In clinical studies, stress has been shown to disrupt the gut microbiota. Under stress, the permeability of the intestinal mucosa increases, α-diversity of the intestinal microbiota increases, and relative abundance of approximately half of the species changes, including the increase in the abundance of the weaker dominant groups, while the more dominant groups, such as *Bacteroides* and lactic acid bacteria, decrease in abundance (Knowles et al., 2008; Karl et al., 2017). In stress-induced animal models, the abundance of the families Prevotellaceae and Coriobacteriaceae and the genus *Prevotella_1* increased significantly, whereas those of Peptococcaceae and Veillonellaceae were significantly reduced (Zhang et al., 2019b). Central stress can alter the transcription of intestinal epithelial genes, such as *Reg3b*, *Duox2*, *Nos2*, and other inflammation-related genes, accompanied by changes in the intestinal microbiota and translocation of bacteria (Allen et al., 2022). Piglets stressed by maternal separation have altered gut microbiota and are more prone to diarrhea, weight loss, and death. Alkaline mineral water can inhibit the HPA axis, thereby reshaping the intestinal microbiota, maintaining the stability of the intestinal epithelium through the brain–microbiota–gut axis, and improving digestive system symptoms (Chen et al., 2022b).

As stated above, antibiotic-induced changes in the maternal microbiota can affect CNS function in offspring, especially that of microglia. Conversely, the central stress of the mother affects the composition of the intestinal microbiota of the offspring while affecting microglia. Clinical data have shown that infants born to mothers with high levels of anxiety, depression, and stress have reduced alpha diversity and a relative abundance of *Bifidobacterium dentalis* in their gut microbiota (Galley et al., 2023). In animal studies, dams exposed to prenatal stress had altered gut microbiota in their female offspring and not in their male offspring and exhibited more severe colonic damage in a model of neonatal necrotizing enterocolitis-like injury. Tissue damage is due to higher microbiota IgA binding in female offspring than in male offspring (Brawner et al., 2020).

Microglia, as important immune cells of the CNS, affect the gut microbiota in several ways. In Ang II-induced hypertension model mice, the number and activation of microglia increased, α-diversity of the intestinal microbiota decreased, and evident clustering appeared (Sharma et al., 2019). Considering the broad physiological functions of microglia in brain development and maintenance of homeostasis, they are thought to be involved in the pathogenesis of various psychiatric and neurodegenerative diseases, which consequently, may affect the gut microbiota. For example, in patients with autism spectrum disorder (ASD), the density of sensitized microglia is increased and the expression of pro-inflammatory genes in microglia is upregulated (Gupta et al., 2014; Lee et al., 2017). Altered gut microbiota with increased *Clostridia* and *Lactobacilli* and decreased *Bacteroides* and *Bifidobacteria* were found in stool samples from children with ASD (Abdel-Haq et al., 2019). AD is another disease in which microglia play an important role. Microglia act as phagocytes in the brain and are responsible for clearing amyloid Aβ (Abdel-Haq et al., 2019). Chronic neuroinflammation and the release of pro-inflammatory factors caused by the activation of microglia are also important causes of AD (Li et al., 2018). AD also disturbs the intestinal microbiota. Increased proportions of *Bacteroidetes*, Firmicutes, Lachnospiraceae, Ruminococcaceae, and Bacteroidaceae have also been observed in patients with AD. In aged AD mouse models, reduced abundance of Firmicutes, Verrucomicrobia, Proteobacteria, and Actinobacteria have been observed (Zhou et al., 2022). Microglia are also involved in the pathogenesis of PD. Studies have shown that abnormally activated microglia affect the intercellular transfer of α-synuclein and promote the progression of PD (George et al., 2019). The composition of the gut microbiota is significantly altered in patients with PD. A clinical study found that the gut microbiota diversity of patients with PD was lower than that in healthy controls. The abundance of Verrucomicrobia, *Prevotella*, *Porphyromonas, Lactobacillus*, and *Parabacteroides* increased, and dysbiosis of the microbiota correlated with intestinal inflammation (Lin et al., 2019). In addition, SCFAs were significantly reduced among the intestinal microbiota metabolites in patients with PD (Unger et al., 2016).

This chapter focuses on correlation studies and lacks the exploration of causality. Diseases and changes in the intestinal microbiota are the result of the joint action of several factors and are not necessarily caused solely by microglial regulation. As there is a two-way connection between the CNS and the GI tract, the role of microglia in shaping the intestinal microbiota is inevitable; however, more research is needed to confirm this. Therefore, we speculated a possible mechanism by which microglia affect the gut microbiota from the perspective of CNS regulatory mechanisms (Fig. 4).

#### The potential mechanism by which the CNS affects the gut microbiota

Activation of the HPA axis under stress is an important pathway by which the CNS and microglia affect the gut microbiota. Stress can lead to intestinal barrier dysfunction, abnormal activation of intestinal inflammation, and ectopic intestinal bacteria, leading to changes in the gut microbiota (Shaler et al., 2021). Mechanistically, the activation of the HPA axis is accompanied by increased levels of circulating CRH and corticosterone. The corticotropin-releasing hormone receptor 1 (CRHR1) mediates colonic inflammation and mucosal damage (Li et al., 2017). Elevated corticosterone levels reduce intestinal permeability, increase systemic inflammation, and change the structure of intestinal microbiota, which is dependent on the existence of GR (Zheng et al., 2013).

Another possible mechanism involves afferent and efferent neural pathways. Cholinergic vagal efferent fibers can stimulate enteric neurons and subsequently inhibit macrophages to release inflammatory cytokines such as IL-1β, IL-6, IL-18, and TNF-α (Agirman et al., 2021). The DRG is a terminal region that originates from the transmission of peripheral sensations (including general somatic and visceral senses) to the brain. The intestinal nociceptor Nav1.8 and transient receptor potential vanilloid 1^+^ (TRPV1^+^) neurons derived from the DRG can mediate the body’s defense against *Salmonella* by regulating the Peyer’s patch microfold cells to change the ileal microbiota structure, abundance, and levels of segmented filamentous bacteria (Lai et al., 2020). The sympathetic nervous system is widely distributed throughout the digestive tract and regulated by the CNS. Postganglionic neurons of the sympathetic nerve can release neurotransmitters, such as norepinephrine and dopamine into the gut lumen. After receiving these neurotransmitters, the commensal and pathogenic microbiota in the digestive tract express virulence genes, accelerate growth, and cause changes in the structure of the microbiota (Mayer et al., 2022) (Fig. 4).

#### Influence of central microglia on the microbiota in IBS

As mentioned above, some brain regions in patients with IBS are activated and the gut microbiota is significantly altered. The microglia and gut microbiota of IBS-like model animals are also disturbed. Visceral hypersensitivity is the most important pathophysiological mechanism of IBS, and some studies have suggested that the pro-inflammatory cytokine granulocyte-colony-stimulating factor released by microglia is a key mediator of visceral sensitization (Basso et al., 2017). Increased visceral sensitivity is markedly attenuated by the inhibition of microglial activation (Zhang et al., 2016; Yuan et al., 2020; Atmani et al., 2022; Ji et al., 2022). Furthermore, directly targeting the P2RY12 purinergic receptor on microglia for blockade can inhibit chronic visceral pain mediated by TRPV1^+^ visceral afferent nerves (Defaye et al., 2022).

Patients with IBS commonly have psychiatric comorbidities. The amelioration of depression-like behaviors in multiple animal models can also be achieved by targeting microglia (Li et al., 2023b; Yang et al., 2023). Minocycline is a microglia-specific inhibitor. Orally administered minocycline in rats with spinal cord injury was found to alleviate cognitive and affective impairments in injured rats and cause changes in the gut microbiota, implying a regulatory function of the gut microbiota of microglia (Schmidt et al., 2021). Minocycline reduces the number of prefrontal microglial cells in a rat model of depression and improves the cecal microbiome by increasing the number of butyric acid-producing bacteria, such as Lachnospiraceae (Schmidtner et al., 2019). However, minocycline is an antibiotic, and whether this change in the gut microbiota is involved remains unknown. Chemically modified tetracycline-3 (CMT-3) is a tetracycline derivative that lacks antibacterial properties, while retaining the inhibitory activity of minocycline on microglia. Intrathecal injection of CMT-3 inhibited microglial activation and it was found to induce a significant antihypertensive effect. This effect is likely mediated by unique changes in the gut microbiota (Sharma et al., 2019).

Although there is no clinical trial evidence at present, we can reasonably speculate that if the microglia of patients with IBS can be inhibited in some way, it is likely to improve the crosstalk between CNS inflammation and the gut microbiota, thereby alleviating their peripheral and central conditions (Fig. 4).

## Prospects for the regulating the interaction between microbiota and microglia in the treatment of DGBIs

The treatment of DGBIs remains a challenge for clinicians, especially for DGBIs with comorbid psychiatric disorders. At present, the main method of treatment is to manage symptoms while considering the psychosocial factors of the patients and treating psychological comorbidities. Tricyclic antidepressants (TcAs) and selective serotonin reuptake inhibitors (SSRIs) are recommended for the treatment of the psychological comorbidities of DGBIs; however, there exist a series of side effects associated with the use of these drugs. Based on the content of this review, interventions targeting the intestinal microbiota to regulate microglia could achieve co-treatment of gut and brain, which is very promising and is a field worthy of further exploration. Limited by the lack of current technological development, there are few studies on the changes of human microglia *in vivo* (Suzuki et al., 2013), and relatively more studies on microglia have chosen to use animal experiments.

**Figure 5.**
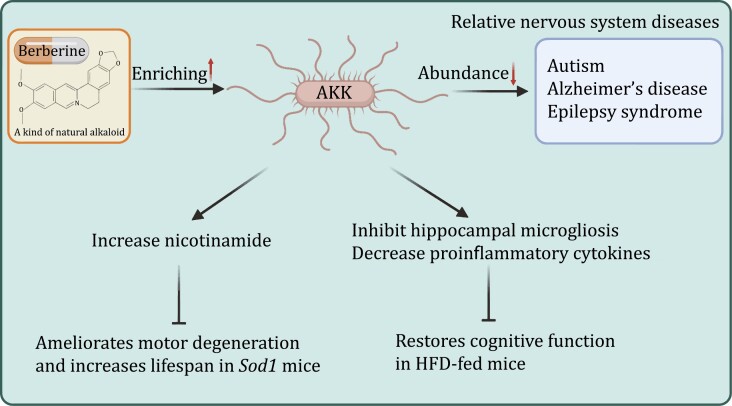
**Potential role of *Akkermansia muciniphila* in the treatment of neurological diseases**. Berberine could significantly enhance the accumulation of AKK in the gut. Altered abundance of AKK in the gut is associated with various neurological diseases. Animal studies have shown that AKK can increase the level of nicotinamide in the brain and alleviate amyotrophic lateral sclerosis. AKK also attenuates HFD-induced cognitive impairment and increases microglia in the hippocampus. AKK, *Akkermansia muciniphila*; HFD, high-fat diet.

Currently, there are many interventions targeting intestinal microbiota. *Roseburia hominis*, a kind of typical probiotics, could inhibit abnormal activation of microglia in GF rats, relieve increased visceral sensitivity, and improve depression-like behavior (Song et al., 2022). Early intervention with a high-fiber diet (rich in prebiotics) reduces PD-like symptoms and brain lesions in α-synuclein-overexpressing ASO mice and upregulates a neuroprotective phenotype of microglia (Abdel-Haq et al., 2022). Similarly, as a typical representative of postbiotics, propionic acid and butyric acid produced by the metabolism of the gut microbiota can also inhibit the activation of microglia (Song et al., 2022). But most of the current research focuses on IBS. Although this has an evident effect, the shortcomings of obvious individual variations have also emerged. In addition, the research and development of some agents is important, similar to the ginsenosides mentioned in this review. BBR, a natural alkaloid, is a highly promising drug. Although clinical studies are lacking, multiple animal experiments provide some evidence. BBR could modulate the intestinal microbiota to alleviate visceral hypersensitivity in WAS-induced IBS mice model. In the IBS mouse model constructed by FMT of IBS patients, BBR also exerted a similar effect, alleviating the increased visceral sensitivity (Zhang et al., 2021). Notably, while improving abdominal symptoms, BBR’s treatment of central inflammation and mental disorders, such as anxiety (Fang et al., 2021) and depression (Huang et al., 2023), has also been partially verified in animal experiments. Our previous studies have shown that BBR intervention can affect the structure and function of the intestinal microbiota, especially the enrichment of *Akkermansia muciniphila* (AKK) and Bacteroidaceae. Recent studies have shown that AKK is related to the occurrence of diseases, such as autism and multiple sclerosis, and can be used as a potential psychobiotic for the next generation (Fig. 5 and Table 2). Further exploration of the potential mechanisms and implementation of clinical trials would be of great significance.

**Table 2.** Potential therapeutic progress of regulating of metabolites or microglia in DGBIs or mental disorders.

Treatment	Subject	Diseases and disorders	Results	References
Probiotics				
*Bacillus coagulans*	Human	IBS	Improving IBS symptom relief rate, as well as global symptom, abdominal pain, bloating, and straining scores.	Zhang et al. (2022a)
Mixed probiotics (*Bifidobacterium longum*, *Lactobacillus acidophilus* and *Enterococcus faecalis*)	Human	IBS	Relief IBS-SSS scale scores in IBS-D patients, relieve abdominal pain symptoms, and reduce plasma MCP-1 and IL-1β levels	Zhang et al. (2019a)
Mixed probiotics (*Bifidobacterium breve* CCFM1025, *Bifidobacterium longum* CCFM687, and *Pediococcus acidilactici* CCFM6432)	Human	Depression	Reduced depression scores (Hamilton Depression Rating, Montgomery-Asberg Depression Rating, and Brief Psychiatric Rating Scales), improved the patients’ gastrointestinal functions (Gastrointestinal Symptom Rating Scale)	Tian et al. (2022)
*Bifidobacterium longum* NCC3001	Human	Depression comorbidity in IBS	Increasing the quality-of-life score and reduce the depression score in IBS patients	Pinto-Sanchez et al. (2017)
Heat-killed *Mycobacterium vaccae*	Mice	Anxiety	Reducing basal levels of genes (*Nlrp3* and *Nfkbia*) involved in microglial priming	Frank et al. (2018)
*Clostridium butyricum*	Mice	PD	Improved motor deficits, loss of dopaminergic neurons, synaptic dysfunction, and microglial activation	Sun et al. (2021)
*Roseburia hominis*	Rat	Depression	Producing SCFAs and inhibits microglia activation	Song et al. (2022)
FMT	Human	IBS	Relief of global IBS symptoms	Johnsen et al. (2018) and El-Salhy et al. (2020)
Prebiotics				
Low-FODMAP	Mice	PD	Inhibiting related pro-inflammatory and neurotoxic signaling pathways, upregulates the neuroprotective phenotype of microglia	Abdel-Haq et al. (2022)
Low-FODMAP	Human	IBS	Altering the composition of intestinal microbiota reduces the concentration of histamine in urine	Staudacher et al. (2014), McIntosh et al. (2017) and Huaman et al. (2018)
Postbiotics				
Propionic acid and butyric acid	Rat	Depression	Inhibiting HDAC1 expression and reduce the expression of various inflammatory factors in the brain	Song et al. (2022)
Butyrate	Mice	Chronic alcoholic central nervous system injury	Improve locomotor hypoactivity, anxiety and depression behaviour, impairs learning, spatial recognition memory, and effectively reduce chronic alcoholic central nervous system damage and correct microglial cell polarization (M1/M2) imbalance	Wei et al. (2023)
NAMO	Mice	HSE	Restoring NAD^+^-dependent mitophagy, inhibiting microglial inflammatory response, and slowing down the progression of HSE	Li et al. (2022a, 2023a)
Drugs				
Rifaximin	Human	IBS	Relief of global IBS symptoms	Lembo et al. (2016)
Shugan granule	Rat	Depression	Regulating the intestinal microbiota, inhibits the activation of microglia and improves intestinal barrier repair	Li et al. (2022c)
Ginsenoside Rh4	Mice	Depression	Alleviating the intestinal microbiota disturbance, inhibiting the excessive activation of microglia and astrocytes, and improve depressive behaviour	Shao et al. (2023)
Berberine	Rat	IBS	Modulating the gut microbiota, alleviates visceral hypersensitivity and reduce the activation of colonic mast cells and spinal cord microglia	Zhang et al. (2021)
Berberine	Rat	Anxiety	Regulating gut microbiota	Fang et al. (2021)
Minocycline	Rat	Depression	Reducing the number of prefrontal microglial cells and improves the cecal microbiome by increasing butyric acid-producing bacteria	Schmidtner et al. (2019)

FMT, fecal microbiota transplantation; FODMAP, fermentable oligosaccharides, disaccharides, monosaccharides, and polyols; HDAC1, histone deacetylase-1; HSE, Herpes Simplex Encephalitis; IBS, irritable bowel syndrome.; IBS-D, IBS with diarrhea; IBS-SSS, IBS symptom severity scale; IL-1β, interleukin-1 beta; MCP-1, monocyte chemoattractant protein-1; NAD, nicotinamide adenine dinucleotide; NAMO, nicotinamide *n*-oxide; PD, Parkinson’s disease; SCFAs, short-chain fatty acids.

## Summary

Microglia play an important role in gut–brain regulation in DGBIs. The gut microbiota is an important regulator of homeostasis, and the regulation and mechanism of the gut–brain axis have received increasing attention. As an important therapeutic target, the regulation of intestinal microbiota may achieve gut–brain co-treatment of DGBI. Given the complexity and inter-individual variability of the gut microbiota, the development of probiotics, prebiotics, synbiotics, and plant drugs that can regulate the intestinal microbiota and potentially affect central microglia, is an important research direction in the future. Restricted by techniques, studies on intestinal microbiota and microglia crosstalk are mainly based on animal models. With the vigorous development of microglia labeling technology and brain functional imaging technology, more studies on brain–gut–microbiota interactions in humans are worth expecting. Furthermore, the mechanism by which microglia affect the gut microbiota is far from being elucidated. It will greatly benefit the understanding and treatment of diseases if future work can uncover more brain–gut mechanisms.

## Abbreviations

Aβ: amyloid-beta
ABX: antibiotic
ACTH: adrenocorticotropic hormone
AD: Alzheimer’s disease
AHR: aryl hydrocarbon receptor
AKK: Akkermansia muciniphila
ASD: autism spectrum disorder
ATP: adenosinetriphosphate
βAR: beta adrenergic receptor
BBB: blood–brain barrier
BCAAs: branched-chain amino acids
BDNF: brain-derived neurotrophic factor
bFGF: basic fibroblast growth factor
CML: *N*6-carboxymethyllysine
CMT-3: chemically modified tetracycline-3
CNS: central nervous system
CRH: corticotropin-releasing hormone
CRHR1: corticotropin-releasing hormone receptor 1
CSF1: colony-stimulating factor 1
CSF1R: CSF1 receptor
CUMS: chronic and unpredictable mild stress
CX3CR1: C-X3-C motif chemokine receptor 1
DAP12: DNAX activation protein of 12 kDa
DGBIs: disorders of gut–brain interaction
EGF: epidermal growth factor
FC: functional constipation
FD: functional dyspepsia
FGIDs: functional gastrointestinal disorders
fMRI: functional magnetic resonance imaging
FMT: fecal microbiota transplantation
FXR: farnesoid X receptor
GF: germ-free
GI: gastrointestinal
GPR43/109A: G protein-coupled receptor 43/109A
GR: glucocorticoid receptor
HDAC1: histone histone deacetylase-1
HFD: high-fat diet
HGF: hepatocyte growth factor
HPA: hypothalamic‒pituitary‒adrenal
HSV-1: herpes simplex virus type 1
IAA: isoamylamine
IBS: irritable bowel syndrome
IBS-D: IBS with diarrhea
IBS-SSS: IBS symptom severity scale
IFN: interferon
IGF1: insulin like growth factor 1
IL: interleukin
IL-3Rα: IL-3 receptor alpha
IL-6R: IL-6 receptor
low-FODMAP diets: low fermentable oligosaccharides, disaccharides, monosaccharides and polyols diets
LPS: lipopolysaccharide
LRRK2: leucine-rich repeat kinase 2
MCP-1: monocyte chemoattractant protein-1
MHC: major histocompatability complex
MMD: maternal microbiota dysbiosis
MMPs: matrix metalloproteinases
mPFC: medial prefrontal cortex
NGF: nerve gowth factor
NLRP3: pyrin-domain-containing 3
PD: Parkinson’s disease
PGE2: prostaglandin E2
PNS: peripheral nervous system
ROS: reactive oxygen species
SCFAs: short-chain fatty acids
SOD1: superoxide dismutase 1
SPF: specific pathogen-free
SSRIs: selective serotonin reuptake inhibitors
TBI: traumatic brain injury
TcAs: tricyclic antidepressants
TGF: transforming growth factor
TGFβR: TGFβ receptor
TGR5: Takeda G protein-coupled receptor 5
TLR: toll-like receptor
TMAO: trimethylamine *N*-oxide
TNF-α: tumor necrosis factor alpha
TREM2: triggering receptor expressed on myeloid cells-2
Trp: tryptophan
TRPV1+: transient receptor potential vanilloid 1+
THIK-1: TWIK-related halothane-inhibited K channel
TWEAK: TNF-associated weak inducer of apoptosis
UTP: uridine triphosphate
WAS: water avoidance stress
